# What Affects Social Attention? Social Presence, Eye Contact and Autistic Traits

**DOI:** 10.1371/journal.pone.0053286

**Published:** 2013-01-09

**Authors:** Megan Freeth, Tom Foulsham, Alan Kingstone

**Affiliations:** 1 Psychology Department, University of Sheffield, Sheffield, United Kingdom; 2 Department of Psychology, University of Essex, Colchester, United Kingdom; 3 Department of Psychology, University of British Columbia, Vancouver, British Columbia, Canada; University of Leicester, United Kingdom

## Abstract

Social understanding is facilitated by effectively attending to other people and the subtle social cues they generate. In order to more fully appreciate the nature of social attention and what drives people to attend to social aspects of the world, one must investigate the factors that influence social attention. This is especially important when attempting to create models of disordered social attention, e.g. a model of social attention in autism. Here we analysed participants' viewing behaviour during one-to-one social interactions with an experimenter. Interactions were conducted either live or via video (social presence manipulation). The participant was asked and then required to answer questions. Experimenter eye-contact was either direct or averted. Additionally, the influence of participant self-reported autistic traits was also investigated. We found that regardless of whether the interaction was conducted live or via a video, participants frequently looked at the experimenter's face, and they did this more often when being asked a question than when answering. Critical differences in social attention between the live and video interactions were also observed. Modifications of experimenter eye contact influenced participants' eye movements in the live interaction only; and increased autistic traits were associated with less looking at the experimenter for video interactions only. We conclude that analysing patterns of eye-movements in response to strictly controlled video stimuli and natural real-world stimuli furthers the field's understanding of the factors that influence social attention.

## Introduction

The world around us contains a vast array of often rapidly changing information. Selectively attending to relevant information helps us to better understand our environment and to make informed judgements on the best future course of action. Attending to social information and learning to interact with others enable us to function successfully in society and so are important skills to develop. However, the factors that influence the manner in which we attend to other living beings, in particular conspecifics (hereafter termed “social attention”) are not fully understood. If we can identify the factors that influence social attention and understand how these factors influence social attention, this will lead to a better understanding of human social behaviour. In recent years interest in analysing attention during natural behaviour has increased [1]–[6] providing the opportunity to gain insights into the subtleties of real-world behaviour, rather than just laboratory-controlled human behaviour. However, to date the majority of social attention research has not been conducted in real-world settings. The vast majority of social attention research has been conducted using computers in laboratory settings in which the social partner is not physically present, see [7]–[9] for reviews. Although extremely valuable in improving knowledge of some of the mechanisms that underlie social attention, this approach may cause researchers to overlook certain factors that influence real world social attention behaviour.

Recent EEG work has suggested that there are fundamental differences in the neural response to viewing another person in the same room compared to viewing that person on a computer screen. Ponkanen et al. [10] found that viewing a live face with direct gaze results in more pronounced neural processing than viewing a photograph of the same face. The presence of another person also influences eye movements in a real world conversation, with gaze becoming coordinated as participants jointly attend to items being discussed [11]. Similar coordination has been reported in a face-to-face referential communication task [12]. Research from social psychology shows that we often avoid looking at other people in real life [13], [14], and this effect has recently been confirmed using an eye-tracking device [15]. In contrast, people, and in particular their faces and eyes, strongly capture and direct attention when participants view photographs [16]–[18]. It therefore seems likely that people may not attend to other individuals in the same way when interacting in real life as when presented with a video. However, to date this has not been tested empirically. Here we compare and contrast allocation of attention during a live face-to-face interaction and during a social interaction presented via a pre-recorded video by analysing eye movements in each type of interaction. In both experiments the task and situation were matched, save for this manipulation. Participants were required to listen to an experimenter's questions and provide verbal responses to the questions, while their eye movements were tracked. In Experiment 1 the experimenter was physically present, sitting in front of the participant, asking questions and then waiting for answers. In Experiment 2 participants were shown a video recording of the experimenter asking the questions and then waiting for the answers the participant provided. This set-up enabled investigation of whether social attention was affected by the experimenter being physically present or not. Using this novel method, it was not possible to test the same participants in each experiment as we wanted each participant to experience the interaction for the first time. Hence social presence was a between-subjects manipulation. However, participants in each experiment were all undergraduate students and therefore from a similar demographic. Other factors that we anticipated may influence social attention were systematically manipulated within-subjects. These factors are outlined in the following paragraphs.

In a social situation the direction of our eyes can be highly informative, for instance when turn-taking during a conversation, see [19] for a review. Looking directly at a speaker while listening can aid decoding of speech as the lips move in synchrony with speech sound [20], and also indicate to the speaker that the listener is attending to what is being said. However, when responding to a question, there is evidence to suggest that averting one's gaze from other people can help us to think more clearly and effectively as this may reduce visual processing demands and cognitive load [21], [22]. Thus it seems likely that social attention would be different depending on whether the current task is listening to another speaker or generating a response. Averting gaze can serve both cognitive and social functions during interactions. Indeed, no association was found between the amount of time participants spent looking at other people's eyes when observing conversations and a general measure of their social skill [23], indicating that socially able individuals do not necessarily spend more time looking directly at other people. Consequently, in the current study we predicted that gaze would be mainly directed away from the experimenter while participants were answering questions in order to reduce cognitive load and facilitate response generation. In addition, an important function of averting gaze whilst speaking in a live conversation may be to provide a social cue to the listener that the speaker has not yet finished providing a response. We therefore anticipated that gaze aversion when answering questions would be particularly pronounced in Experiment 1, the live interaction, as in this experiment averting gaze would also serve a social function, indicating to the experimenter that the response to the question was not yet complete. This cue would be unnecessary when responding to a video and so we might expect reduced gaze aversion in Experiment 2.

One of the other factors that we anticipated would affect social attention is eye contact. Eye contact provides a foundation for communication and social interaction [24] and modulates activation of the social brain network [25]. Direct eye contact is visually captivating, and even young infants prefer to look at faces with direct gaze than averted gaze [26]. Being able to maintain eye contact, but also to modulate gaze appropriately, are both important for social development [25], [27]. There is thought to be automatic and rapid detection of other individuals making eye contact with an observer. Indeed, recent evidence actually suggests that there is enhanced unconscious representation of direct gaze compared to averted gaze [28]. In the current study experimenter gaze direction was systematically manipulated in order to investigate the effect that the conversant's gaze direction had on social attention. The experimenter looked directly at the participant's face during two of the questions and averted her gaze down towards her notes during the other two questions. Comparing the effect of experimenter eye contact between a face-to-face interaction and a pre-recorded interaction indicates whether modifications of social attention relating to eye contact are influenced by social presence. If eye contact indeed creates a reciprocal social signal during conversation, it is likely that any effect of experimenter eye contact on participant eye movements will be more pronounced in the live interaction.

Individuals with certain neurological disorders have been shown to express difficulties with maintaining and modulating social attention effectively. For example, individuals with damage to the amygdala exhibit a severe reduction in direct eye contact during conversations [29]. For individuals with Autism Spectrum Disorder (ASD) one of the defining, and indeed most striking, features is “unusual eye-contact” [30]. A tendency to look less at faces has been reported in infants between 9 and 12 months of age, who later received a diagnosis on the autism spectrum [31], [32]. In addition, toddlers with ASD generally preferred to look towards dynamic geometric patterns than videos of other children [33], and young children with autism (mean age 4 yrs 8mnths) looked significantly less at faces than did typically developing children when viewing video-taped conversations [34]. However, evidence from eye tracking studies conducted with older individuals with ASD presents a more mixed picture. Some studies report that individuals with ASD do not fixate on other people's faces as much as typically developing controls when presented with social stimuli, [35]–[37]. However, reduced fixations on people, especially their faces, has not been universally observed in adolescents and adults with autism, e.g. [23], [38]–[40], indicating that reduced social attention in autism is not a ubiquitous finding. Rather, a complex mixture of factors leads to the unusual social attention profile observed in autism (see also [41], for a discussion of ASD and alexythimia). Of particular relevance to the current paper is a study by Nadig et al. [42] who recorded eye-movements of a group of pre-adolescents with high-functioning autism (HFA) and a group of control participants during conversations with a social partner on generic topics, and on topics of particular interest to the participant. No differences were observed between groups in overall time spent looking at the face of the social partner. However, within the HFA group when discussing generic topics, there was an inverse relationship between severity of autistic symptoms and time spent looking at their partner's face, such that participants with HFA who looked less to their partner's face displayed more autistic symptoms. In addition, a study very recently published by Noris et al. [43] found that in a naturalistic interaction with an adult experimenter, young children with ASD spent significantly less time looking directly at the experimenter's face than did their typically developing peers. However, as far as we are aware, to date these are the only published studies which have used eye-tracking technology to examine eye-movements during a live social interaction in individuals with autism. Consequently, the relationship between the nature of eye-movements in real world social interactions and the severity of autistic symptoms is currently not well understood.

An indication of autistic traits in the typically developing population can be obtained by administering the Autism-spectrum Quotient [44]. It has recently been shown that when direct eye contact is delivered from a person in a video, observers who score highly on the AQ are more likely to look away from the eyes than low AQ scorers [45]. Here we investigated whether the amount of autistic traits an individual possesses correlates with social attention behaviour in a live interaction and an interaction presented via video. In line with recent research findings [45], we predicted that there would be a negative correlation between AQ score and total time spent fixating the experimenter's face in the interaction presented via video. In the naturalistic live interaction our predictions were less clear. We anticipated that the physical presence of a person may increase the intensity of the social situation and so may also increase the strength of this correlation resulting in an even stronger negative relationship between autistic symptoms and time spent looking at the experimenter in the live interaction, cf. [42]. Conversely, as this study involves individuals from the typically developing population, pressure to adhere to ‘social norms’ may be created in the live interaction resulting in reduced looking time towards the experimenter by participants overall, cf. [15], and perhaps especially so in more socially aware participants, i.e. those who have less autistic traits. This may result in dissolution of the relationship between autistic traits and time spent looking at the experimenter in the live interaction.

In summary, the experiments reported in this paper aimed to investigate factors that influence social attention during an interaction and how these factors interact. Specifically, the influence of social presence, current task – listening to or answering a question, eye contact and autistic traits were investigated. Participants' eye movements during one-to-one question-and-answer social interactions with an experimenter were analysed. Interactions were either conducted live (Experiment 1) or via video (Experiment 2), a between-subjects manipulation of social presence. Interactions were split into “Ask” phases, in which the participant listened to a question being asked, and “Answer” phases, in which the participant gave a verbal response to the question asked. Experimenter eye gaze was either directed towards the participant's face or averted downwards, a within-subjects manipulation of eye contact. The potential influence of amount of participant self-reported autistic traits on social attention was also investigated. The observation of similar effects between Experiment 1 and Experiment 2 would encourage greater confidence in research that uses video presentation as an experimental substitute for real life situations. We predicted that participants would look more at the experimenter while listening to questions as this would likely aid decoding of speech as the lips move in synchrony with speech sound, cf. [20]. Additionally, averting gaze away from the experimenter while answering questions may serve an attempt to reduce cognitive load. We predicted that this effect would be accentuated in the live interaction as this may also serve as a social cue to the experimenter that the participant has not yet finished speaking. We also predicted that differences in participant eye movements when the experimenter made direct eye contact compared to averting gaze away from the participant would be more pronounced in the live interaction, due to the physical presence of a person which enhances the social nature of the interaction. For reasons outlined in the previous paragraph, no firm prediction on the nature of the relationship between autistic traits and time spent looking at the experimenter in the live condition was made. However, we anticipated that there would be a negative relationship between autistic traits and time spent looking at the experimenter in the video interaction, cf. [45]. This series of analyses served to aid understanding of the mechanisms involved in social attention and highlight any potential differences in the nature of social attention between real world interactions and a pre-recorded presentation of similar visual and auditory stimuli.

## Experiment 1 – Patterns of Eye Movements during a Live, Face-to-Face, Interaction

### Method

#### Participants

Thirty-two undergraduate students participated in this experiment. Sixteen participants were undergraduate students studying at the University of British Columbia, Canada. Sixteen participants were recruited from the University of Essex, UK (participants were recruited from two different testing sites due to the experimenter changing institutions rather than for any theoretical reason). Participants provided full written informed consent prior to participating in the study. One participant from the University of Essex was excluded as no eye-tracking data recorded during one of the questions, resulting in a final sample size of 31 participants (16 male; 15 female). Mean AQ score = 16.6, SD = 5.4 (range: 9–30). Due to participants being recruited from two different sites, we initially ran all of the analyses (see below) on the data from each site separately. Trends for all main effects and interactions reported below were also found independently in each sample. The nature of the correlation analyses also did not differ between samples. Below we report analyses on the combined the samples. Local ethics approval was obtained from the institutions involved.

#### Apparatus and Materials

Participants at the University of British Columbia wore an Applied Science Laboratory Mobile Eye eye-tracker which consists of a head-mounted system built into a pair of glasses and a small, portable recording device. A scene camera, coinciding with the participant's line of sight, recorded the scene in front of the person with a field of view of about 50° (horizontal) by 40° (vertical). Pupil and corneal reflections were recorded monocularly from the video image of the right eye at 30 Hz. Point of regard was then superimposed over the scene image as a circular cursor, allowing measurement of what was being looked at in each frame of the recorded video. Participants at the University of Essex wore a similar SMI-HED system (SensoriMotoric Instruments). This system also records the scene, along with pupil and corneal reflections (at 50 Hz) from cameras fitted to a headwear, resulting in a video with a gaze cursor formatted in exactly the same way as the ASL eye-tracker. Both mobile eye trackers have an instrumental spatial resolution of approximately 0.1° and yield typical gaze position accuracy of 0.5°–1°. Calibrations were achieved and validated by asking participants to fixate a series of points on a board spanning the central visual field and at the same distance as the interviewer.

Viewing locations were coded frame by frame by a researcher using in-house software, run through MATLAB. This researcher was blind to the AQ scores and gender of participants. The software presented the location of gaze for each frame and the coders recorded whether the centre of the gaze cursor was located on one of the three regions of interest: “face/head”, “body” and “background”. “Background” included all regions that were not the experimenter's face/head or body. For frames which no cursor was present, due to blinks, out of range looks or eye-tracking loss, no location of gaze code was assigned. Proportion of viewing time on each region was calculated by dividing the sum of coded frames for each location by the total sum of coded frames. With the equipment used, and because in the no eye contact condition the head was tilted down and the eyes were often not visible, it was not possible to make further conclusions regarding the relative importance of different facial features. A pseudo-random selection of 25% of data was coded by a second independent coder who was blind to all participant details and experimental hypotheses. The percentage of frames on which the two coders agreed was calculated, yielding inter-rater reliability of 96.88%.

All participants completed the Autism-spectrum Quotient (AQ) questionnaire. Scores on this 50 item self-report questionnaire provide an indicator of the degree to which an individual possesses traits associated with the autistic spectrum. The Baron-Cohen et al. [44] collapsed scoring method was used: responses in the “autistic” direction were given a score of 1, and responses in the “non-autistic” direction were given a score of 0. Participants therefore received a score between 0 and 50, higher scores indicating the presence of more autistic traits. In the original article [44], male students scored an average of 18.6; female students scored an average of 16.4. A clinically significant score on the AQ is 32 – in the original article 80% of individuals with ASD scored at or above this level compared to 2% of individuals without a diagnosis of ASD.

#### Procedure

**Figure 1:**
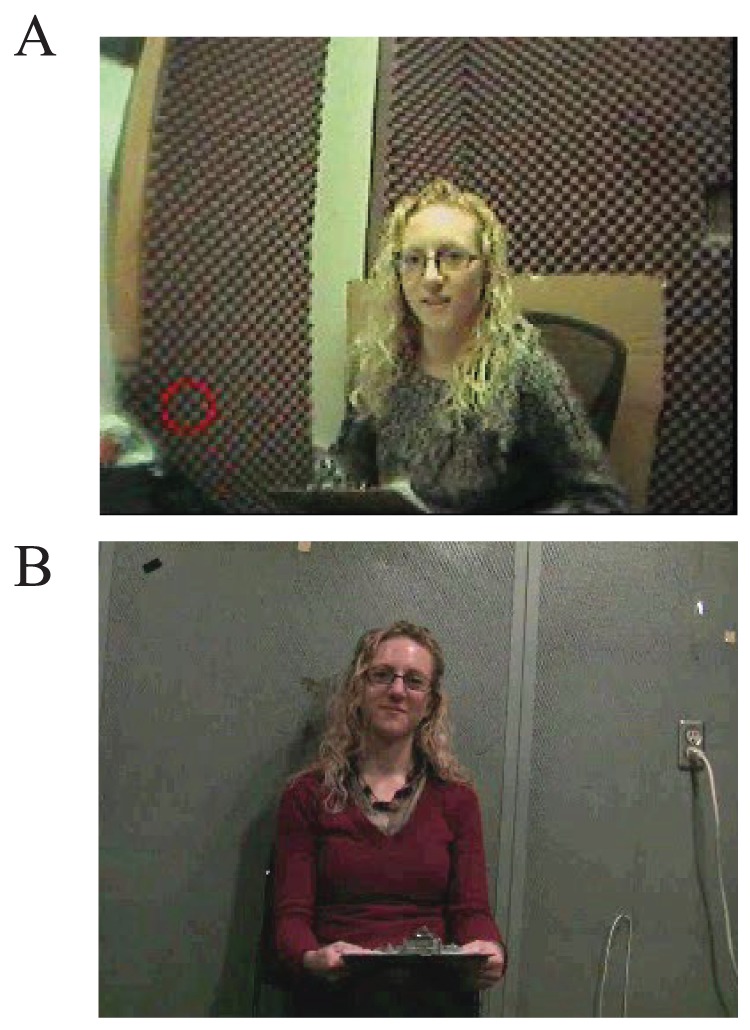
Examples of participant views of the experimenter in A: Experiment 1 - live interaction. Red circle indicates fixation location. B: Experiment 2 - video-taped interaction.

Each participant completed a one-to-one interaction with an experimenter. Participants were given the following instruction: “I'm going ask you to talk about four topics that you'll need to discuss whilst your eye movements are tracked. There is a microphone on the eye tracker that will record your answers.” Participants were then fitted with the mobile eye-tracker. The female experimenter sat across the desk from the participant, approximately 1 metre away (see Figure 1a). Participants' eye movements and verbal responses were recorded. The average duration of verbal response was 28.75 s (long answers were cropped at 30 seconds so that each participant contributed a similar amount of data). Each participant was made aware that they could move their head if they so wished. Therefore they were free to look at the experimenter as much of as little as they liked, or to look at the wall, other objects in the background or turn away.

All participants were asked to talk about a series of four topics. The experimenter looked directly at the eyes of the participant (direct eye contact condition) while asking about two of the topics and listening to the answers. The experimenter looked down at her notes for the remaining two topics (no eye contact condition). The topics were presented in the same order to each participant. However, topics for which eye contact was made were counterbalanced between participants to ensure that experimenter gaze direction was independent of topic and order (topics are outlined in Table 1).

**Table 1:** Interaction topics.

Topic number	Topic
1.	Tell me some things you like about living in Vancouver and some things you dislike about living in Vancouver
2.	Tell me about some things that you did last weekend and some things that you plan to do next weekend
3.	Describe a few things you consider to be typically Canadian and a few things you consider to be typically American
4.	Tell me about some things you do in your spare time; then pick one sport or activity of your choice and either describe some of the rules or tell me how you would go about doing that sport or activity

Following the interaction participants were asked to complete the Autism-spectrum Quotient (AQ) self-report questionnaire.

### Results

Each question was split into an Ask phase (i.e. while the experimenter was asking the question) and an Answer phase (i.e. the first 30 seconds of the participant's verbal response). The viewing locations recorded for each frame in which a cursor was present (face or head; body; background) were used to calculate the proportion of total viewing time on each region during each phase. All main variables were checked for outliers (values greater than 2 SD from the mean) resulting in one participant being removed from further analysis, leaving a final dataset of 30 participants.

A 2×3×2 repeated measures ANOVA (Question phase (Ask/Answer)×Region (Face/Body/Background)×Eye contact (Direct/Averted)) on proportion of viewing time was conducted, the results of which are organised by topic below.

#### Region of Interest Analysis

There was a main effect of region, *F*(2,58) = 21.01, *p*<.001, *η_p_^2^* = 0.42. Post-hoc *t*-tests, using Bonferroni corrected alpha levels of 0.016, indicated that participants looked more at the experimenter's face than body overall, *t*(29) = 6.84, *p*<.001, *d* = 2.00, despite the body occupying more of the visual field; there was no significant difference between the amount of time spent looking at the face and the amount of time spent looking at the background overall, *t*(29) = 0.06, *p* = .95, *d* = 0.04, again despite the background occupying far more of the visual field. Participants looked significantly more at the background than at the experimenter's body, *t*(29) = −8.36, *p*<.001, *d* = 2.06.

#### The Effect of Question Phase on Viewing Behaviour

**Figure 2:**
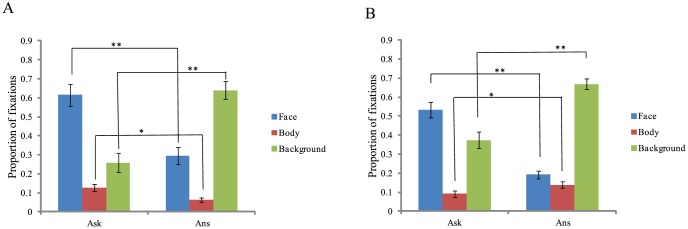
Location and proportion of viewing time in each question phase A: Experiment 1- live interaction. B: Experiment 2 - video-taped interaction. ** p<.001 *p<.05.

Because the proportions in each question phase summed to 1, a main effect could not be observed. There was a significant interaction between question phase and region, *F*(2,58) = 51.76, *p*<.001, *η_p_^2^* = 0.64, as participants distributed their viewing time differently when being asked a question compared to answering. Post-hoc *t*-tests, using Bonferroni corrected alpha levels of 0.016, indicated that participants looked more at the experimenter's face in the Ask phase than the Answer phase, *t*(29) = 6.87, *p*<.001, *d* = 1.11. Participants looked more at the experimenter's body in the Ask phase than the Answer phase, *t*(29) = 3.35, *p* = .002, *d* = 0.79, and participants looked less at the background in the Ask phase than the Answer phase, *t*(29) = −7.87, *p*<.001, *d* = 1.43 (see Figure 2a). These results demonstrate that, in support of our hypotheses, participants averted their gaze away from the interviewer while answering questions compared to when listening to the questions being asked.

#### The Effect of Experimenter Eye Contact on Viewing Behaviour

**Figure 3:**
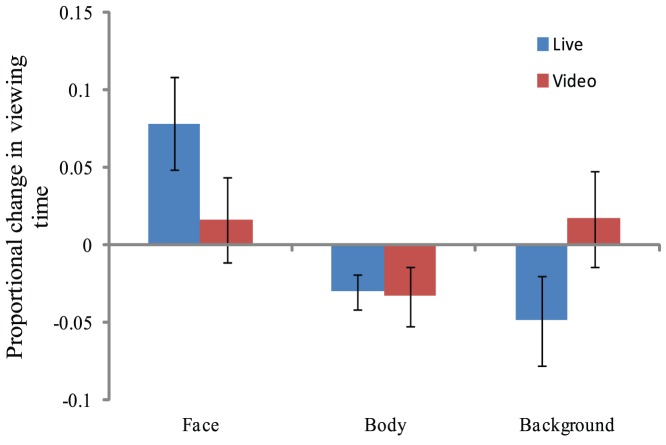
Change in viewing time to each region of interest with eye contact (Eye contact – no Eye contact), for Live and Video experiments.

The direction of the experimenter's gaze, either directed at the participant's face or averted, influenced viewing behaviour as indicated by a significant eye contact×region interaction, *F*(2,58) = 5.07, *p* = .009, *η_p_^2^* = 0.15. Figure 3 shows the change in mean viewing time as a function of eye contact (i.e., eye contact minus no eye contact). Post-hoc *t*-tests using Bonferroni corrected alpha levels of 0.016, indicated that participants looked more at the experimenter's face when eye contact was made, *t*(29) = 2.60, *p* = .014, *d* = 0.27, and participants looked less at the experimenter's body when eye contact was made, *t*(20) = 2.71, *p* = .011, *d* = 0.38. Although there was a complementary trend for participants to look less at the background when eye contact was made, this did not reach significance, *t*(29) = 1.69, *p* = .10, *d* = 0.16.

There was also an eye contact×region×question phase interaction, *F*(2,58) = 4.63, *p* = .014, *η_p_^2^* = 0.14. Two post-hoc 2×3 ANOVAs were run on the question phase data and answer phase data separately. It was found that the effect of modification of experimenter gaze direction (eye contact vs. no eye contact) was large and significant in the Answer phase, *F*(2,58) = 9.99, *p*<.001, *η_p_^2^* = 0.26. However the effect was smaller and non-significant in the ask phase, *F*(2,58) = 2.18, *p* = .12, *η_p_^2^* = 0.07, indicating that the overall eye contact×region interaction was driven by viewing behaviour in the Answer phase.

As the experimenter was always female we wanted to check whether any of the effects interacted with gender. Gender was added as an additional between subjects factor into the ANOVA reported above. Gender did not interact with any factor, or combination of factors (all *p*>.05).

#### The Effect of Autism-spectrum Quotient scores on Viewing Behaviour

Multiple bivariate Pearson's correlations were conducted to investigate whether participants' AQ scores were related to viewing patterns. Data met parametric assumptions of normality and homogeneity of variance. AQ score did not correlate with proportion of time spent looking at the experimenter (the experimenter's face/head+body), *r*(29) = .04, *p* = .82. To ensure that AQ score was not interacting with other factors manipulated, further correlational analyses were run. There was no hint of a relationship between AQ score and proportion of time spent looking at the experimenter in the direct eye contact condition in either the Ask phase, *r*(29) = .04. *p* = .84, or the Answer phase, *r*(29) = .11, *p* = .55; neither was there any relationship when the experimenter averted gaze in either the Ask phase, *r*(29) = .03. *p* = .89, or the Answer phase, *r*(29) = −.03, *p* = .87. There were no statistically outlying scores in these analyses (more than 2 SD from the mean). From this series of analyses we can conclude that AQ score was not related to viewing patterns in the live interaction. It is worth noting that not only were these correlations all non-significant for a live social interaction, they were also mainly in the opposite direction to the effect reported by Chen and Yoon [45], who observed that increased autistic traits were associated with looking less at a face on a video presentation.

### Discussion

Experiment 1 found that participants spent a large proportion of their viewing time directing their attention at their social partner, especially looking at the experimenter's face, even though the experimenter's face occupied only a small portion of the visual field. Participants were much more likely to look at the experimenter when being asked a question than when giving an answer and they were especially likely to look at the face when being asked a question. In this live interaction scenario the eye gaze direction of the experimenter affected participants' viewing patterns significantly. Overall, it was found that participants looked more at the experimenter's face and less at the experimenter's body when direct eye contact was made. The experimenter's eye gaze direction had a stronger effect on participant's viewing patterns in the answer phase than in the ask phase. These results indicate that direct gaze in a live situation is a powerful social cue, cf. [10]. AQ scores did not correlate with proportion of viewing time spent looking at the experimenter overall. In addition, AQ did not correlate with time spent looking at the experimenter as a function of eye contact, and this was true in both the Ask and Answer phases. Our results demonstrated a different pattern of results to those observed by Chen and Yoon [45] who presented participants with videos of face stimuli and observed a positive relationship between gaze aversion and AQ score. Contrary to our suggestion that a real social interaction may increase the strength of this effect, our findings suggest that additional factors influence behaviour when face stimuli are observed in live face-to-face interactions, thus eliminating the relationship between AQ and looking behaviour. We anticipated that the nature of the results from Experiment 2 which presented a pre-recorded video to participants would be closer to those observed by Chen and Yoon [45]. If so, this would indicate that there is something inherently different in experiencing a live interaction, in which there is a real possibility of influencing the experimenter's thoughts or behaviour, compared to an interaction presented via video.

## Experiment 2 – Patterns of Eye Movements during a Pre-recorded Video Interaction

### Method

#### Participants

Thirty-two undergraduate students, who had not taken part in Experiment 1, participated in this experiment (16 males; 16 females). All participants were recruited through the University of British Columbia Human Subject Pool. They gave their full written informed consent and received course credit in return for participating. One female participant's data had to be removed due to poor calibration. Therefore, the dataset contained 31 participants, mean AQ score = 19.6, SD = 5.2 (range: 7–32). The study was approved by the University of British Columbia's Ethics Board.

#### Apparatus

Participants were presented with a video on a 19 inch monitor at a viewing distance of 23 in, which resulted in an image approximately 40°×31° of visual angle. Eye movements were recorded using the EyeLink II eye-tracking system which uses a head mounted camera. Pupil position was recorded monocularly from the video image of the right eye at 500 Hz, with spatial accuracy of at least 0.5°. Following a 9-point on screen calibration, the system used an online parser to extract fixations and saccades based on velocity (30/°s) and acceleration (8000°/s^2^) thresholds. The coordinates of each fixation were then compared to rectangular areas of interest encompassing the face and body of the experimenter, which were the same size throughout the video condition. Sound was played through a pair of speakers positioned on either side of the monitor. In the video condition participants spoke their answers into a microphone that was attached to the eye tracker, and the responses were stored on computer as a digital sound file.

#### Procedure

A female experimenter was videotaped from a distance of approximately 1 metre; asking each of the four questions and waiting for an answer (see Figure 1b). These videos were presented on a monitor and the eye-tracked participant was asked to speak his or her answers into the microphone after each question. During this response a video of the experimenter waiting was played, and the participant terminated this waiting period when the answer was completed by pressing a key. As in Experiment 1, eye movements from the first 30 seconds of each answer were analysed. The experimenter did not fill the visual field and, as before, the wall and objects in the background were also available to be looked at. In other words, each participant was free to look at the experimenter as much or as little as they liked (see Figure 1b).

All participants were asked to talk about the four topics used in Experiment 1. The experimenter looked straight into the camera, simulating eye contact with the participant while asking them about two of the topics and listening to the answers. The experimenter looked down at her notes for the remaining two topics. As in Experiment 1, topics were presented in the same order to each participant. However, topics for which eye contact was made were counterbalanced between participants to ensure that topic content was independent of experimenter gaze direction. Similarly, following the question and answer interview, participants were asked to complete the AQ self-report questionnaire.

### Results

As in Experiment 1 each question was split into an Ask phase (i.e. while the experimenter was asking the question) and an Answer phase (i.e. the first 30 seconds of the participant's verbal response). The viewing locations recorded for each frame (face or head; body; background) were used to calculate the proportion of total viewing time on each region during each phase. All main variables were checked for outliers (values greater than 2 SD from the mean) resulting in one participant being removed from further analysis, leaving a final dataset of 30 participants.

A 2×3×2 repeated measures ANOVA (Question phase (Ask/Answer)×Region (Face/Body/Background)×Eye contact (Direct/Averted)) on proportion of viewing time was conducted, the results of which are organised by topic below.

#### Region of Interest Analysis

As for the live interactions, there was a main effect of region, *F*(2,58) = 38.62, *p*<.001, *η_p_^2^* = 0.57. Post-hoc *t*-tests, using Bonferroni corrected alpha levels of 0.016, indicated that participants looked more at the experimenter's face than body overall, *t*(29) = 7.43, *p*<.001, *d* = 1.8, despite the body occupying more of the visual field. In contrast to Experiment 1, in Experiment 2 participants looked more at the background than the experimenter's face overall, *t*(29) = 2.65, *p* = .013, *d* = 0.9. Participants looked significantly more at the background than at the experimenter's body, *t*(29) = 9.55, *p*<.001, *d* = 2.9.

#### The Effect of Question Phase on Viewing Behaviour

There was an interaction between question phase and region, *F*(2,58) = 105.45, *p*<.001, *η_p_^2^* = 0.78, as participants distributed their viewing time differently when being asked a question compared to answering. Post-hoc *t*-tests, using Bonferroni corrected alpha levels of 0.016, indicated that participants looked more at the experimenter's face in the Ask phase than the Answer phase, *t*(29) = 11.08, *p*<.001, *d* = 1.9; participants looked less at the experimenter's body in the Ask phase than the Answer phase, *t*(29) = 3.12, *p* = .004, *d* = 0.5; and participants looked less at the background in the Ask phase than the Answer phase, *t*(29) = 10.62, *p*<.001, *d* = 1.6 (see Figure 2b). These results demonstrate that, in support of our hypotheses, participants averted their gaze away from the experimenter's face while answering questions compared to when listening to the questions being asked, though in contrast to Experiment 1 the proportion of looking time on the experimenter's body increased slightly when answering.

#### The Effect of Eye Contact on Viewing Behaviour

Unlike Experiment 1, the direction of the experimenter's gaze, either directed at the participant's face or averted, did not significantly influence which regions participants looked at, indicated by a non-significant eye contact×region interaction, *F*(2,58) = 0.78, *p* = .46, *η_p_^2^* = 0.03 (Figure 3). Also in contrast to Experiment 1, there was no eye contact×region×question phase interaction, *F*(2,58) = 0.02, *p* = .98, *η_p_^2^* = 0.001. Taken together these results indicate that the direction of experimenter gaze direction had no influence on participants viewing behaviour during this video based interaction.

As in Experiment 1 gender did not interact with any factor, or combination of factors (all *p*>.05).

#### Comparison of Viewing Behaviour between Experiment 1 and Experiment 2

In order to investigate whether the nature of viewing behaviour differed significantly between the live interaction and the pre-recorded video interaction, an omnibus ANOVA was conducted to directly compare the two experiments. A 2×2×3×2 mixed measures ANOVA (between-subjects factor of Experiment×Question phase×Region×Eye Contact) found no interaction between experiment and region, *F*(2,116) = 2.26, *p* = .11, *η_p_^2^* = .04, indicating that the difference in how proportion of viewing time was distributed between regions in Experiment 1 and Experiment 2 did not reach significance. However, there was an Experiment×Region×Question phase interaction that approached significance, *F*(2,116) = 2.96, *p* = .06, *η_p_^2^* = 0.05. Considering the Ask phase and Answer phase separately, the magnitude of the Experiment×Region interaction brushed significance in the Answer phase, *F*(2,116) = 2.99, *p* = .05, *η_p_^2^* = 0.05 but was non-significant in the Ask phase, *F*(2,116) = 2.09, *p* = .13, *η_p_^2^* = 0.04 suggesting that the difference in viewing behaviour between experiments was larger in the Answer phase than the Ask phase. A specific difference that we predicted between experiments was that gaze aversion away from the experimenter's face while answering questions would be more pronounced in Experiment 1 compared to Experiment 2. In order to test this hypothesis, an independent samples *t*-test on the proportion of time spent looking at the experimenter's face in the Answer phase in each experiment was conducted. Contrary to our hypothesis it was found that a greater proportion of viewing time was allocated to the experimenter's face in the Answer phase in Experiment 1 than Experiment 2, *t*(58) = 2.11, *p* = .04, *d* = 0.5

Regarding the potential difference in the effect of eye contact between experiments, a 3-way Eye contact×Region×Experiment interaction did not reach significance, *F*(2,116) = 2.07, *p* = .13, *η_p_^2^* = 0.3. In addition a 4-way Eye contact×Region×Question phase×Experiment interaction did not reach significance, *F*(2,116) = 2.03, *p* = .14, *η_p_^2^* = 0.3. Yet in Experiment 1 modulation of experimenter eye-contact only affected viewing behaviour relating to the experimenter's face and body, with direct eye contact increasing significantly the proportion of time spent looking at the face and reducing significantly the proportion of time spent looking at the body (no significant effect on proportion of viewing time on the background was observed). We were therefore interested to know whether there were statistical differences in the effect of eye contact on proportion of time spent viewing the face and body of the experimenter between experiments. Therefore, a 2×2×2×2 mixed measures ANOVA (Experiment×Question phase (Ask/Answer)×Region (Face/Body)×Eye contact (Direct/Averted)) was conducted. This analysis returned a 3-way Eye contact×Region×Experiment interaction that brushed significance, *F*(1,58) = 4.15, *p* = .05, *η_p_^2^* = 0.07, indicating that the nature of the interaction between viewing behavior on the experimenter and eye contact was statistically different in each experiment. There was also a 3-way Eye contact×Question phase×Experiment interaction that brushed significance, *F*(1,58) = 4.17, *p* = .05, *η_p_^2^* = 0.07, suggesting that the interaction between question phase and eye contact on viewing behavior on the experimenter was different in each experiment. Taken together these findings demonstrate that modulation of experimenter eye contact had a greater effect on how participants viewing behaviour in the live interaction (Experiment 1) than in the pre-recorded interaction (Experiment 2), as can be seen in our description for each experiment above.

#### The Effect of Autism-spectrum Quotient scores on Viewing Behaviour

Multiple bivariate Pearson's correlations were conducted to investigate whether participants' AQ scores were related to viewing patterns. Data met parametric assumptions of normality and homogeneity of variance. AQ score was found to negatively correlate with proportion of time spent looking at the experimenter (the experimenter's face/head+body), *r*(29) = −.37, *p* = .05. Low AQ scorers – individuals with fewer autistic traits - were more likely to look at the experimenter than the High AQ scorers – individuals with more autistic traits. There were no statistically significant outlying scores (more than 2 SD from the mean). The relationship between AQ score and proportion of time spent looking at the experimenter was evident when considering trials in which the experimenter made eye-contact; Ask phase, *r*(29) = −.36, *p* = .05, Answer phase, *r*(29) = −.46, *p* = .01, but there was no significant relationship when eye-contact was not made; Ask phase, *r*(29) = −.16, *p* = .39, Answer phase, *r*(29) = −.23, *p* = .22.

### Discussion

Some of the results observed in the video interactions replicated the patterns observed in the live interactions. Participants spent a large proportion of their viewing time directing their attention at their social partner, especially looking at the experimenter's face. This was despite the experimenter's face occupying only a small portion of the visual field. As in Experiment 1 participants were more likely to look at the experimenter when being asked a question than when giving an answer, and in particular, they were more likely to look at the face when being asked a question. However, in this video interaction scenario modifications of experimenter eye gaze direction did not influence participants' viewing patterns. This was strikingly different from the effects observed in the live interactions, in which modifications to experimenter eye gaze direction influenced participants' viewing patterns. Also in contrast to the results of the live interaction, AQ scores correlated negatively with proportion of viewing time on the experimenter overall (i.e. individuals with more autistic traits looked less at the experimenter). It seems that, like individuals with ASD, participants with more autistic traits tend to look at other people less when presented with video stimuli. The results suggest that direct gaze in particular may be avoided. An important methodological point to note is that different individuals participated in each experiment. Confidence in the robustness of the differences in social attention observed between experiments could have been increased further had the same participants completed each experiment. However, as we were keen that each participant should experience the interaction for the first time, a within-subjects design was not possible.

## General Discussion

The present experiments sought to investigate the factors that influence social attention during an interaction, and to discover whether these factors interact with each other. Specifically, the influence of social presence, current task (listening to a question being asked or answering a question), eye contact and autistic traits were examined. Interactions were either conducted live (Experiment 1) or via a pre-recorded video presentation (Experiment 2). Each interaction was split into “Ask” phases, as the experimenter asked each question, and “Answer” phases, as the participant answered. Experimenter eye contact was either directed towards the participant's face or averted downwards, a within-subjects manipulation of eye contact. The potential influence of amount of participant self-reported autistic traits on social attention was also investigated. Certain patterns of viewing behaviour emerged both when individuals were presented with a real life social interaction and when they were presented with the same scenario via video. We therefore conclude that these effects were independent of social presence. The similar effects were as follows: 1) Overall, participants looked at the experimenter (face/head plus body) for a similar proportion of viewing time in both experiments. 2) Participants looked at the experimenter more when being asked a question compared to when answering. 3) Participants were more likely to look at the experimenter's face than body – even though the body took up a much greater proportion of the visual array. 4) Participants looked at the experimenter's face significantly less when answering a question compared to listening to it being asked. These similarities between live and video interactions demonstrate that many aspects of eye movement behaviours generalize between natural and artificial stimuli. This highlights the research value of using video presentation stimuli in a controlled laboratory situation.

However, differences between patterns of eye movements in the live interaction and the video interaction were also observed, indicating that certain effects were influenced by social presence. The following specific differences were observed: 1) Modifications in experimenter eye gaze direction had a significant effect on participants' eye movements in the live interaction only; participants looked more at the experimenter's face and less at her body and the background when eye contact was made compared to when her gaze was averted. No such effect was observed in the video interactions. 2) Modification in experimenter eye contact had a pronounced and significant effect in the answer phase of the live interaction but only a modest and non-significant effect in the ask phase of the live interaction, suggesting that viewing behaviour in the Answer phase is most sensitive to experimenter eye contact, perhaps because in this phase there is the most scope for influencing the behaviour of the social partner. No effects of experimenter eye gaze direction were observed in the video interactions. 3) In the video interaction only, AQ score correlated with viewing patterns. This effect was particularly prominent when the experimenter simulated eye contact. There were no correlations between AQ score and proportion of viewing time in the live interactions. These differences between viewing patterns in Experiment 1 and Experiment 2 demonstrate that one must exhibit caution when attempting to draw parallels between experiments using video and those featuring real interactions, and highlights the necessity of conducting research both in controlled and natural environments.

The finding that participants looked less at the experimenter's face while answering questions than listening to them being asked may be due to participants using the visual cues of the experimenter's moving lips to help them decode the speech. This reduced looking at the face during the answer phases also supports Doherty-Sneddon et al.'s findings [21], [46] that participants tend to avert their gaze when they are thinking of answers to questions when sitting in front of the person asking the questions in an attempt to reduce cognitive load. We predicted that gaze aversion while answering questions would be stronger in the live interaction as it may also provide a communicative signal to the experimenter that the participant is still in the process of producing an answer and not yet ready to receive the next question. However, contrary to our hypothesis it was found that a greater proportion of viewing time was allocated to the experimenter's face in the Answer phase of the live interaction than the video interaction. Non-verbal communication to a conversant is therefore unlikely to be the only reason for averting gaze while answering. Perhaps fixating on an observed person's face distracts resources from the central task of answering the question. Our finding that participants actually looked at the social partner significantly more when answering a question in the live interaction than in the video interaction may be due to perceived social expectation. It might be that participants feel they should look at a social partner while in the presence of that partner out of politeness or positive impression management and this prevents participants from averting their gaze to a greater extent.

Experimenter eye contact was shown to affect viewing patterns in the live interaction but not in the video interaction. It seems that the effect of experimenter eye contact was greater in the live interactions and more effective at capturing participants' attention. Confidence in this finding could have been increased further had the same participants completed both experiments. However, these data suggest that there is something inherently social about eye contact that affects behaviour when another person is present in the room and there is the possibility for modifying the listener's behaviour, thus creating a reciprocal social signal. The results of the present study demonstrate that attempting to devise models of social attention on the basis of studies conducted in the laboratory alone may be at best of limited utility and at worst misleading. The future challenge will be to discover *why* these differences occur. One interesting scenario in this respect concerns video conferencing or video chat over the internet. This scenario provides the potential for manipulating the social response of another person but lacks the physical presence of the person in the same room. The cognitive ethology approach to research, proposed by Kingstone, Smilek & Eastwood [47] provides a framework for future investigation into these real world effects that may otherwise be missed. Collecting behavioural data in both standard laboratory settings and real-world environments will greatly expand our knowledge of the factors that influence cognition and social attention in particular.

In the current study an association between more autistic traits and a smaller proportion of viewing time on a person when watching a video was observed. This replicates much of the autism literature reporting that individuals with autism tend to look at people less when watching videos [33]–[36], [41], but see [23]. Our finding also supports Chen and Yoon's report [45] that this effect extends to the broader autism phenotype, within the typically developing population. In contrast to findings from the interactions conducted via video, the present study found no correlation between autistic traits and proportion of viewing time on the experimenter in the live interaction. Confidence in the differing relationship between autistic traits and viewing behaviour in each experiment could have been increased further had the same participants completed both experiments. However, the absence of a simple relationship between increased autistic traits and less overall time spent looking at the experimenter in the live interaction is consistent with results reported by Nadig et al. [42] who did not observe reduced looking at a social partner during conversation in individuals with High Functioning Autism (HFA), though an inverse relationship between severity of autistic symptoms and time spent looking at a partner's face was observed within the HFA group. It is possible that in a real world interaction, with a physically present conversant, social cues such as eye contact are so strong that they overcome any abnormalities in social attention (e.g. a lack of interest in social information) that are present in those with autistic traits. Conversely, it may be that rules relevant to social norms are applied when another person is physically present in the room, norms that are less relevant when observing a pre-recorded person speaking/listening. For example, one knows that in real life it is not socially acceptable to stare at a person as this will alter their judgement of us [13]–[15] whereas when observing a pre-recorded video, this potential for judgement by the observed person is absent. Perhaps the more socially aware individuals – the Low AQ scorers – are more affected by this distinction, so their tendency to fixate an observed person's face more than High AQ scorers was diminished in the live interaction situation. In the more complex, naturalistic setting it might be that both high and low AQ scorers decreased their attention to the experimenter but for different reasons—a reduced sensitivity to the social stimulus of a face (for high AQ scorers) and a keener appreciation of social norms (for low AQ scorers). However, there is an alternative explanation for the negative correlation between AQ score and fixations on the experimenter being present in the video experiment only. Perhaps to individuals with Low autistic traits another person's face, especially direct gaze, is extremely captivating. It may be that individuals with High autistic traits do not experience the same captivation with a video presentation of an observed person; perhaps this type of stimulus is inherently less interesting to these individuals. In future work it will be of empirical and theoretical importance to establish which of these alternative explanations is correct.

The results from the current study highlight that differences relating to autism - or autistic traits - may be misleading if data collected from laboratory settings is the only information used to understand the cognitive profile related to social attention. For instance, an intervention strategy, often used when attempting to enhance the social skills of individuals with autism, is to tell them to “look at the eyes”. It is worth considering that in doing so, a potentially abnormal strategy is being encouraged. As noted by Norbury et al. [23], who found no association between time spent looking at the eyes and social skill, more is not necessarily better in terms of making eye contact with another person. From the current study and other related work in this area e.g. [4], [11], [12], [14], [15], it is clear that the subtleties of social attention are much more complex than merely staring at the eyes of another individual. Indeed, the wide variation in proportion of viewing time spent looking at the experimenter within our sample demonstrates this. Improving our knowledge of the factors that influence naturally occurring social attention will enable better intervention strategies to be developed to assist individuals, such as those with ASD, who have difficulties in this key domain.

It is important to consider the possibility that some of the observed differences between experiments could have been caused by factors other than those that these experiments were designed to manipulate. For example, in the live interaction participants were able to move their head and even to move their upper body if they so wished as they were wearing a mobile eye-tracker. Thus it was possible for participants to lean slightly forward or back thus changing the angle within the visual array covered by the experimenter. Our results demonstrated that overall participants spent a greater proportion of their viewing time looking at the background than the experimenter's face in the video interaction whereas they spent as much time looking at the face as they did looking at the background in the live interaction. Participants could have leant forward in the live interaction, thus causing the experimenter to occupy a greater proportion of their visual field; the flip side being that participants in the live condition could also have chosen to turn their heads away from the experimenter entirely. However, it seems more likely that participants simply found the live interaction more engaging and therefore did not avert their gaze away from the experimenter as much in Experiment 1 as Experiment 2. The main differences that we found between the two experiments related to experimenter eye contact and self-reported autistic traits. We do not believe that any incidental differences between the two experimental environments could have led to the observed differences in relation to these factors.

The results of the current study have demonstrated that considering experimental context is extremely important when designing social attention experiments. Certain aspects of viewing patterns were consistent across both the live interaction and the video interaction, which were closely matched in terms of audio and visual input to the participant. However, experimenter eye contact and participants' self-reported autistic traits had a different effect in each situation. The results of this study demonstrate that caution should be exercised when using only a video presented stimulus as a substitute for real life. Directly comparing the two scenarios can provide insights into social attention that would not be possible if either were considered in isolation.
